# Unraveling the causal link: fatty acids and inflammatory bowel disease

**DOI:** 10.3389/fimmu.2024.1405790

**Published:** 2024-07-25

**Authors:** Yi Zhou, Zhenhua Zhou

**Affiliations:** Department of General Surgery, Medical Center of Digestive Disease, Zhuzhou Hospital Affiliated to Xiangya School of Medicine, Central South University, Zhuzhou, China

**Keywords:** Mendelian randomization, fatty acids, inflammatory bowel disease, ulcerative colitis, Crohn’s disease, causal association

## Abstract

**Background:**

Previous observational studies have revealed the strong relationship between fatty acids (FA) and inflammatory bowel disease (IBD). Nonetheless, due to the inherent limitations of retrospective research, the causality between the two has not been clearly established.

**Methods:**

Genetic variants associated with the 17 FA indicators were derived from genome-wide association studies. Summary statistics for the discovery cohort and testing cohort for IBD, including ulcerative colitis (UC) and Crohn’s disease (CD), were available from IIBDGC and FinnGen, respectively. Bidirectional MR analysis and sensitivity analysis with multiple measures were applied to comprehensively investigate the causal link between FA and IBD.

**Results:**

Combining the results of various MR methods, the validation of testing cohort, and the merging of meta-analysis, we demonstrated that genetically predicted Omega-3 FA levels, Ratio of Omega-3 FA to total FA, Docosahexaenoic acid (DHA) levels, and Ratio of DHA to total FA reduced the risk of IBD, UC, and CD. Meanwhile, multivariate MR suggested that the risk effects of Omega-3 FA and DHA for UC and CD were mainly affected by Saturated FA and Monounsaturated fatty acid (MUFA). Furthermore, although there was the causal association between Ratio of MUFA to total FA as well as Ratio of Polyunsaturated fatty acid (PUFA) to MUFA and CD, sensitivity analysis prompted that the findings were not robust. None of the above results had a reverse causal effect.

**Conclusion:**

This MR investigation provided evidence of causality between diverse FA and IBD. These findings offered new insights into the treatment and prevention of IBD.

## 1 Introduction

Inflammatory bowel disease (IBD) is divided into two basic subtypes, ulcerative colitis (UC) and Crohn’s disease (CD), and is known as a chronic, immune-mediated, and nonspecific inflammatory disease of the intestinal tract (1). The prevalence of IBD has exceeded 0.3% among many countries in North America, Europe, and Oceania, and its incidence is escalating worldwide (2). Abdominal pain, recurrent diarrhea, weight loss, and a few systemic symptoms are among the clinical signs of IBD (3). In addition, the main treatments for IBD encompass the use of immunosuppressants and immunomodulators (4). At present, the pathogenesis of IBD is not entirely clear, but the factors of immunity, environment, genetics and gut microbiology have been suggested to be tightly linked (5). Patients with IBD have high occupancy of healthcare resources, significantly reduced quality of life, and suboptimal treatment efficacy. Furthermore, the existence of IBD may promote the development of many autoimmune diseases and malignancies at the same time, which further aggravates the public health burden (6, 7). Therefore, it is crucial to seek means to prevent IBD through an understanding of the pathogenesis for this category of illnesses.

Fatty acids (FA) are a set of carboxylic acids with aliphatic chains, which are commonly subdivided into saturated fatty acid (SFA), monounsaturated fatty acid (MUFA), and polyunsaturated fatty acid (PUFA) depending on the degree of unsaturation (8). PUFA are further categorized as Omega-3 or Omega-6 FA based on the position of the first double bond of the terminal methyl group. Linoleic acid (LA) and alpha-linolenic acid (ALA) are two commonly occurring PUFA that can be gained via the diet and act as precursors. With elongation and desaturation, LA is transformed into arachidonic acid (AA), which belongs to the Omega-6 FA. ALA is converted to eicosatetraenoic acid (DPA), docosahexaenoic acid (DHA) and eicosapentaenoic acid (EPA), which classified as Omega-3 FA. In either form, FA are the essential source of energy in the human diet as well as the important structural component of cells (9). Prior research has established that the intake of FA in the diet has been found to modulate gut mucosal inflammation, which may further contribute to the development of IBD (10). The majority of studies investigating the association between FA and IBD are retrospective, which tend to have inherent limitations such as confounding and reverse causation. Thus, it seems unclear whether the correlations reported in these observational studies are causative (11, 12). Moreover, it is hard to execute randomized controlled trials owing to high costs and ethical concerns.

To further surmount the issues mentioned above, Mendelian randomization (MR) furnishes an alternative approach to scrutinize causal effects. MR capitalizes on single nucleotide polymorphisms (SNPs) as instrumental variables (IVs) for exposure to minimize confounding and retain causality, thereby bolstering causal inference about the link between exposures and outcomes (13). In accordance with the Mendelian laws of inheritance, genetic variants are randomly allocated at the time of fertilization and precede any disorders, making the results less susceptible to environmental confounding and reverse causality (14). Consequently, the objective of the study was to systematically elucidate the causal relationships between 17 different circulating FA indicators (levels or ratios) and IBD (UC and CD) through MR.

## 2 Materials and methods

### 2.1 Study design

**Figure 1:**
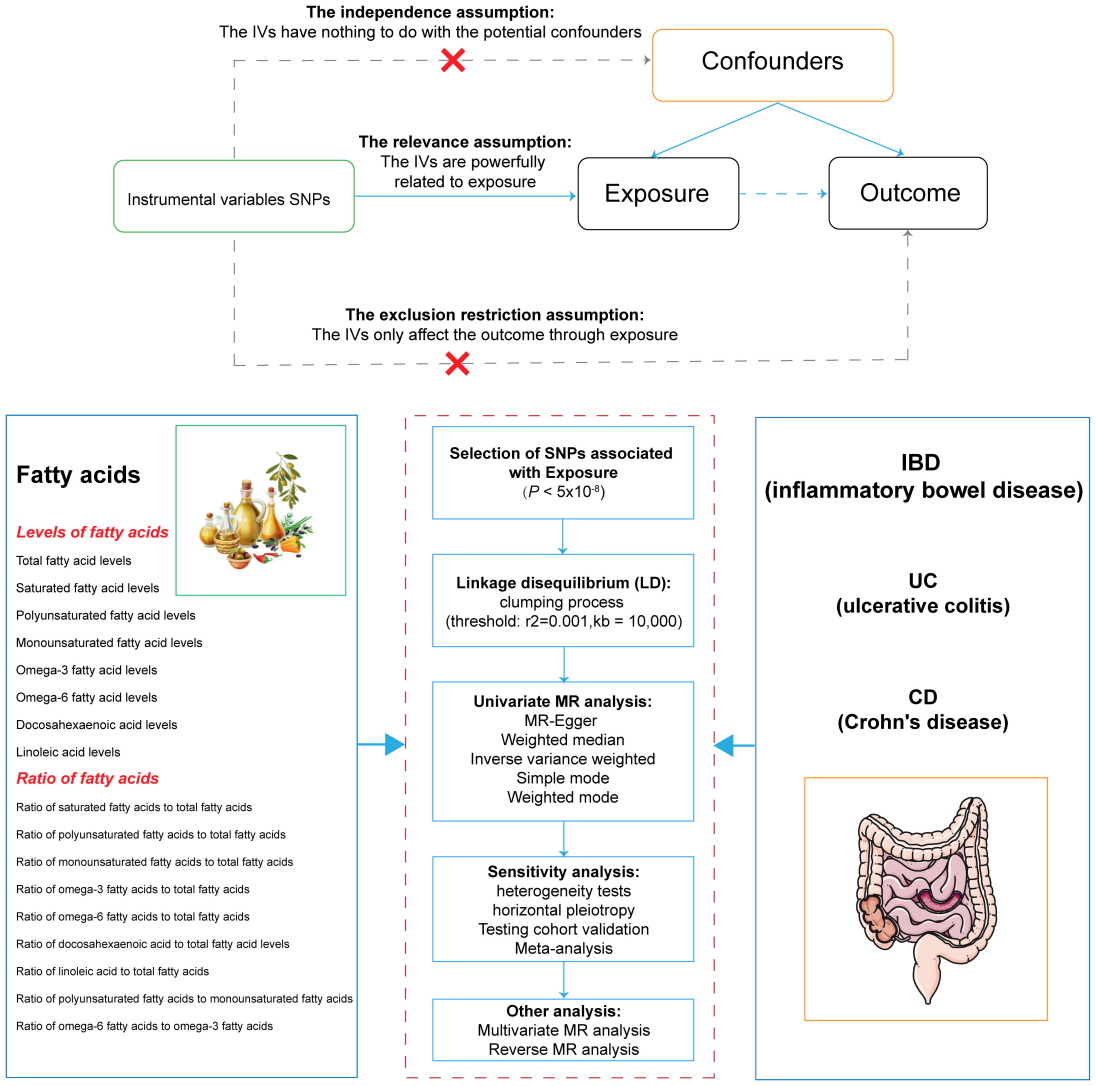
Overview of the design and methods used in this Mendelian randomization study. IVs, instrumental variable; SNPs, single nucleotide polymorphisms; LD, linkage disequilibrium; MR, Mendelian randomization; IBD, inflammatory bowel disease; UC, ulcerative colitis; CD, Crohn’s disease.

The STROBE-MR statement for reporting MR studies was adhered to by our research (15). Using MR techniques, the underlying causative effects of 17 FA markers on IBD and its two subtypes were examined. Three key presumptions underpinned MR’s analysis (16): (1) IVs were strongly correlated with exposure; (2) confounding variables shouldn’t have an impact on IVs; and (3) IVs influenced outcomes exclusively through exposure. This study did not require informed consent or ethical approval because it used publicly available data. **Figure 1** displayed the design of our investigation.

### 2.2 Data sources of FA

We collected summary statistics for 17 distinct circulating FA indicators from the genome-wide association study (GWAS) reported by Richardson TG et al, which contained eight FA level categories and nine FA ratio categories (17). In addition, data related to these FA indicators were standardized and adjusted for gender, age, and the first 10 principal components.

### 2.3 Data sources of IBD and its subtypes

GWAS summary statistics for IBD and its two subtypes (UC and CD) were drawn from two cohorts of European ancestry, including one discovery cohort and one testing cohort, respectively. Of these, GWAS summary statistics for the discovery cohort were obtained from the IIBDGC, which included 12,882 IBD cases, 6,968 UC cases, and 5,956 CD cases (18). Additionally, GWAS data for the testing cohort were sourced from the FinnGen consortium (https://www.finngen.fi/en), which consisted of 5,673 IBD cases, 4,320 UC cases, and 2,056 CD case. During the analysis, both cohorts adjusted for genetic associations of IBD and its two subtypes on the basis of age, sex, and primary genetic components.

### 2.4 Selection of genetic instruments

Stringent quality control procedures were deployed to filter the optimal IVs (13, 19). SNPs having significance levels across the genome (*P* < 5 × 10^-8^) were extracted. Meanwhile, SNPs with larger physical distances (≥ 10,000 kb) and smaller probability of linkage disequilibrium (R2 < 0.001) were enrolled. Furthermore, the F-statistic of each SNP was exploited to evaluate the strength of the correlation between IV and exposure, preventing the bias imposed by weak IV (20). Therein, weak IV was deemed unbiased when the F-statistic was greater than 10.

### 2.5 Univariate MR analysis

The inverse variance weighted (IVW) method was adopted as the main analysis approach for univariate MR to estimate the association between relevant FA indicators and the risk of IBD (21). Moreover, we used multiple methods to validate and complement the IVW results. First, the consistency of the results was checked using six additional MR analysis methods, including weighted median, MR-Egger, weighted mode, simple mode, MR-robust adjusted profile score (RAPS), and MR-Pleiotropy Residual Sum and Outlier (PRESSO). With at least 50% of the selected IVs valid, the weighted median produced unbiased estimates (22). Under the influence of pleiotropy, MR-Egger regression could be utilized to get the convincing causal effects (23). Although the simple mode was not as powerful as IVW, it provided robustness to pleiotropy (24). The weighted mode approach was inferior in its ability to detect causal effects, but also had less bias (25). Owing to the existence of pleiotropy, the MR-PRESSO method was used to identify outlier SNPs. After removing these outliers, recalculations were performed to gain more accurate causal estimates (26). MR-RAPS proposed an asymptotic mean estimator by adapting the contour scores to boost the robustness and efficiency (27). Second, the results were validated with the testing cohort. Meanwhile, the results of the discovery cohort and the testing cohort were merged using meta-analysis (28). Finally, sensitivity analysis was undertaken to gauge the robustness of the MR results. Horizontal pleiotropy was assayed using MR-Egger regression (23) and MR-PRESSO (26). Heterogeneity with each exposure-related SNP was evaluated with Cochran’s Q test (29).

### 2.6 Multivariate MR analysis

Multivariate MR (MVMR), an extension of standard univariate MR, was an emerging approach to incorporate the genetic variation of each potential confounder or mediator into the same model, which could demonstrate the potential genetic overlap between a given FA indicator and other risk factors that might account for the polymorphism in question (30, 31). Given that different FA indicators might co-exist in the human body, we utilized the MVMR approach to elucidate whether other FA indicators would influence the statistically significant causal effect in the univariate MR.

### 2.7 Reverse MR analysis

In order to assess the impact of genetic susceptibility to IBD, UC and CD on different FA indicators, we also performed the reverse MR analysis.

### 2.8 Statistical analysis

R program (Version 4.3.1) was used for all statistical analysis. The causal association between FA and IBD was analyzed using the R package “TwoSampleMR” (version 0.5.8). Statistical significance was defined as two-sided *P* < 0.05. Moreover, the false discovery rate (FDR) was used to adjust for the multiple testing assumption (32). Of these, causal associations with FDR < 0.05 were considered strong evidence, whereas associations with FDR ≥ 0.05 and *P* < 0.05 were deemed to be the suggestive causal associations.

## 3 Results

### 3.1 Data sources and IV selection

Seventeen FA indicators were captured as exposures in the MR analysis, including eight FA level categories and nine FA ratio categories. The eight different FA level categories consisted of total FA levels, SFA levels, MUFA levels, PUFA levels, Omega-3 FA levels, Omega-6 FA levels, DHA levels, and LA levels. In addition, the nine FA ratio categories comprised Ratio of SFA to total FA, Ratio of MUFA to total FA, Ratio of PUFA to total FA, Ratio of Omega-3 FA to total FA, Ratio of Omega-6 FA to total FA, Ratio of DHA to total FA, Ratio of LA to MUFA, and Ratio of Omega-6 FA to Omega-3 FA. The number of SNPs ranged from 28 to 72. For all 17 FA indicators inspected, the F-statistic for their respective IVs was greater than 10, suggesting no potential weak instrumental bias (**Supplementary Table S1**). Moreover, IIBDGC and FinnGen, which included patients with IBD, UC, and CD, were picked as the discovery cohort and testing cohort for outcomes, respectively. **Table 1** displayed the details of datasets for FA and IBD.

**Table 1:** Description of the contributing GWAS.

Traits	GWAS ID	Consortium/Author	Year	PMID	Sample size	No of cases	No of controls	No of SNPs	F statistics	Ancestry
Exposure (Levels of fatty acids)										
Total fatty acid levels	ebi-a-GCST90092987	Richardson TG et al	2022	35213538	115,006	NA	NA	61	30 to 883	European
Saturated fatty acid levels	ebi-a-GCST90092980	Richardson TG et al	2022	35213538	115,006	NA	NA	54	30 to 655	European
Polyunsaturated fatty acid levels	ebi-a-GCST90092939	Richardson TG et al	2022	35213538	115,006	NA	NA	65	30 to 702	European
Monounsaturated fatty acid levels	ebi-a-GCST90092928	Richardson TG et al	2022	35213538	115,006	NA	NA	72	30 to 1,172	European
Omega-3 fatty acid levels	ebi-a-GCST90092931	Richardson TG et al	2022	35213538	115,006	NA	NA	55	30 to 6,663	European
Omega-6 fatty acid levels	ebi-a-GCST90092933	Richardson TG et al	2022	35213538	115,006	NA	NA	64	30 to 713	European
Docosahexaenoic acid levels	ebi-a-GCST90092816	Richardson TG et al	2022	35213538	115,006	NA	NA	46	30 to 4,997	European
Linoleic acid levels	ebi-a-GCST90092880	Richardson TG et al	2022	35213538	115,006	NA	NA	55	31 to 638	European
Exposure (Ratio of fatty acids)										
Ratio of saturated fatty acids to total fatty acids	ebi-a-GCST90092981	Richardson TG et al	2022	35213538	115,006	NA	NA	30	30 to 177	European
Ratio of polyunsaturated fatty acids to total fatty acids	ebi-a-GCST90092941	Richardson TG et al	2022	35213538	115,006	NA	NA	50	31 to 857	European
Ratio of monounsaturated fatty acids to total fatty acids	ebi-a-GCST90092929	Richardson TG et al	2022	35213538	115,006	NA	NA	69	30 to 2,297	European
Ratio of omega-3 fatty acids to total fatty acids	ebi-a-GCST90092932	Richardson TG et al	2022	35213538	115,006	NA	NA	37	30 to 8,977	European
Ratio of omega-6 fatty acids to total fatty acids	ebi-a-GCST90092935	Richardson TG et al	2022	35213538	115,006	NA	NA	57	30 to 736	European
Ratio of docosahexaenoic acid to total fatty acid levels	ebi-a-GCST90092817	Richardson TG et al	2022	35213538	115,006	NA	NA	28	30 to 4,348	European
Ratio of linoleic acid to total fatty acids	ebi-a-GCST90092881	Richardson TG et al	2022	35213538	115,006	NA	NA	41	31 to 2,005	European
Ratio of polyunsaturated fatty acids to monounsaturated fatty acids	ebi-a-GCST90092940	Richardson TG et al	2022	35213538	115,006	NA	NA	60	30 to 1,712	European
Ratio of omega-6 fatty acids to omega-3 fatty acids	ebi-a-GCST90092934	Richardson TG et al	2022	35213538	115,006	NA	NA	35	31 to 8,028	European
Outcome (IBD-discovery cohort)										
IBD	ieu-a-31	IIBDGC	2015	26192919	34,652	12,882	21,770	NA	NA	European
UC	ieu-a-32	IIBDGC	2015	26192919	27,432	6,968	20,464	NA	NA	European
CD	ieu-a-30	IIBDGC	2015	26192919	20,883	5,956	14,927	NA	NA	European
Outcome (IBD-testing cohort)										
IBD	finn-b-K11_IBD	FinnGen	2021	36653562	218,792	5,673	213,119	NA	NA	European
UC	finn-b-K11_ULCER	FinnGen	2021	36653562	214,620	4,320	210,300	NA	NA	European
CD	finn-b-K11_CROHN	FinnGen	2021	36653562	212,356	2,056	210,300	NA	NA	European

GWAS, genome wide association study; PMID, PubMed unique identifier; IBD, inflammatory bowel disease; UC, ulcerative colitis; CD, Crohn’s disease; NA, not applicable.

### 3.2 Causal effects of FA and IBD

**Figure 2:**
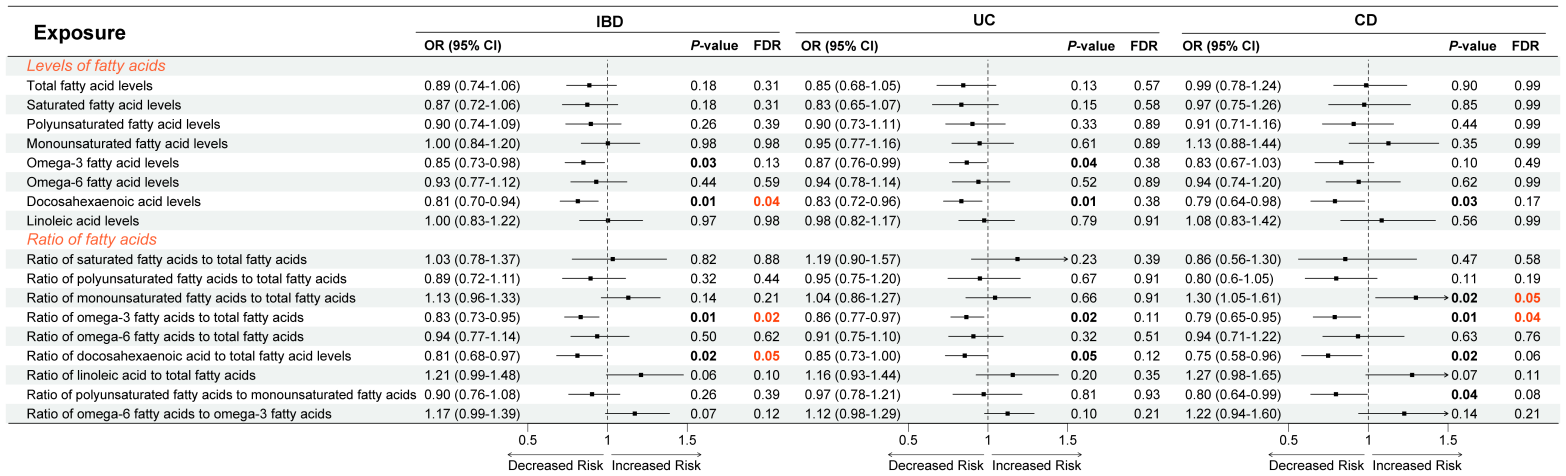
Causal relationship between fatty acids and inflammatory bowel disease using inverse variance weighted method. OR, odds ratio; CI, confidence interval; FDR, false discovery ratio; IBD, inflammatory bowel disease; UC, ulcerative colitis; CD, Crohn’s disease.

With IVW as the main method in univariate MR, **Figure 2** exhibited the causal effects between different circulating FA indicators and IBD, UC, and CD. First, an increase in DHA levels [OR (odds ratio) = 0.81, *P* = 0.01, FDR = 0.04], as well as an increase in Ratio of Omega-3 FA to total FA (OR = 0.83, *P* = 0.01, FDR = 0.02) and Ratio of DHA to total FA (OR = 0.81, *P* = 0.02, FDR = 0.05) were significantly associated with decreased risk of IBD, whereas elevated levels of Omega-3 FA (OR = 0.85, *P* = 0.03, FDR = 0.13) had only a suggestive causal association with reduced risk of IBD (**Figure 2**). Second, elevated Omega-3 FA levels (OR = 0.87, *P* = 0.04, FDR = 0.38) and DHA levels (OR = 0.83, *P* = 0.01, FDR = 0.38), as well as increased Ratio of Omega-3 FA to total FA (OR = 0.86, *P* = 0.02, FDR = 0.11) and Ratio of DHA to total FA (OR = 0.85, *P* = 0.05, FDR = 0.12) had only suggestive causal effects with decreased UC risk (**Figure 2**). Finally, decreased in Ratio of MUFA to total FA (OR = 1.30, *P* = 0.02, FDR = 0.05) and increased in Ratio of Omega-3 FA to total FA (OR = 0.79, *P* = 0.01, FDR = 0.04) were significantly linked to decreased CD risk. In addition, decreased DHA levels (OR = 0.79, *P* = 0.03, FDR = 0.17), as well as increased Ratio of DHA to total FA (OR = 0.75, *P* = 0.02, FDR = 0.06) and Ratio of PUFA to MUFA (OR = 0.08, *P* = 0.04, FDR = 0.08) presented the suggestive causal association with the CD risk reduction. The results of heterogeneity and horizontal pleiotropy were shown in **Supplementary Table S2**.

**Figure 3:**
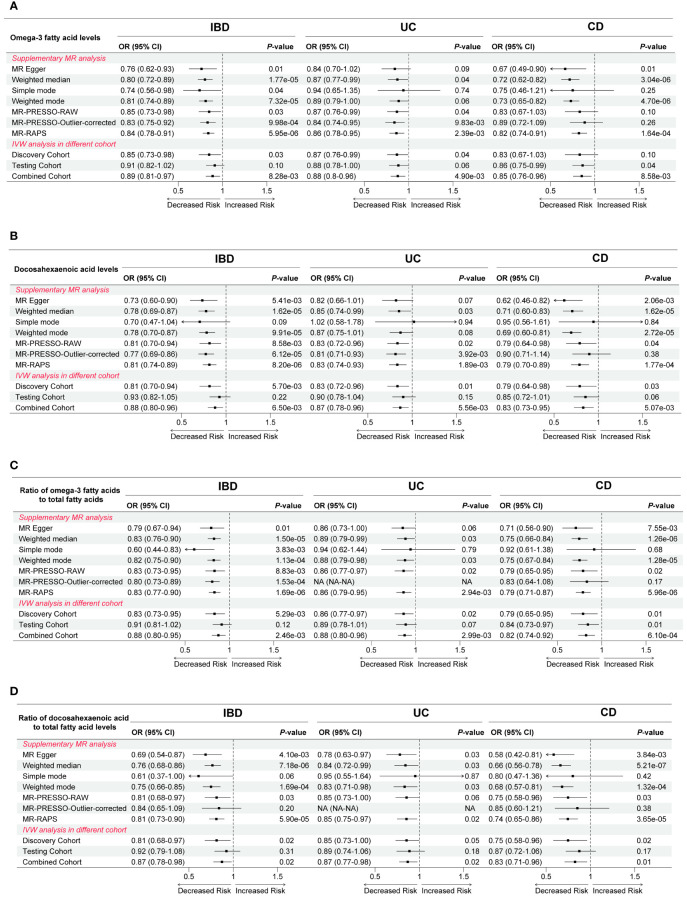
Complementation and validation of causal effects between **(A)** Omega-3 fatty acid levels, **(B)** Docosahexaenoic acid levels, **(C)** Ratio of omega-3 fatty acids to total fatty acids, as well as **(D)** Ratio of docosahexaenoic acid to total fatty acid levels and inflammatory bowel disease and its two subtypes. OR, odds ratio; CI, confidence interval; IVW, inverse variance weighted; PRESSO, Pleiotropy Residual Sum and Outlier; RAPS, robust adjusted profile score; IBD, inflammatory bowel disease; UC, ulcerative colitis; CD, Crohn’s disease.

To guarantee reliability and robustness, we supplemented and validated the IVW results employing a variety of means (**Supplementary Tables S3**-**S6**). First, upon supplementation using six additional MR analysis methods, we found that the results of the associations between Omega-3 FA levels, DHA levels, Ratio of Omega-3 to total FA, and Ratio of DHA to total FA with IBD, UC, and CD were generally consistent (**Figure 3**). Notably, although IVW methods suggested that the correlation between Omega-3 FA levels and CD risk was not statistically significant (OR = 0.83, *P* = 0.10), yet MR Egger, weighted median, weighted mode, and MR-RAPS indicated that elevated Omega-3 FA levels were able to reduce the risk of CD (OR < 1, *P* < 0.05; **Figure 3A**). Moreover, the conclusion that the increase in Ratio of DHA to total FA and Ratio of PUFA to MUFA led to higher risk of CD was also basically robust (**Supplementary Figure S1**). Second, when validated using the testing cohort, there were no statistically significant differences in the association results between the relevant exposures and outcomes, except for the causal effect of the two Omega-3 FA indicators with CD (*P* < 0.05; **Figure 3**, **Supplementary Figure S1**). Finally, when combining the discovery cohort and the testing cohort using meta-analysis, we again validated the IVW results that increases in Omega-3 FA levels, DHA levels, Ratio of Omega-3 to total FA, and Ratio of Omega-3 to total FA, would cause the decreased risk of IBD, UC, and CD (OR < 1, *P* < 0.05; **Figure 3**, **Supplementary Table S6**). However, the correlation between Ratio of DHA to total FA as well as Ratio of PUFA to MUFA and CD lost statistical difference (*P* > 0.05; **Supplementary Figure S1**).

### 3.3 Impact of independent and co-existing FA indicators on MR results

**Figure 4:**
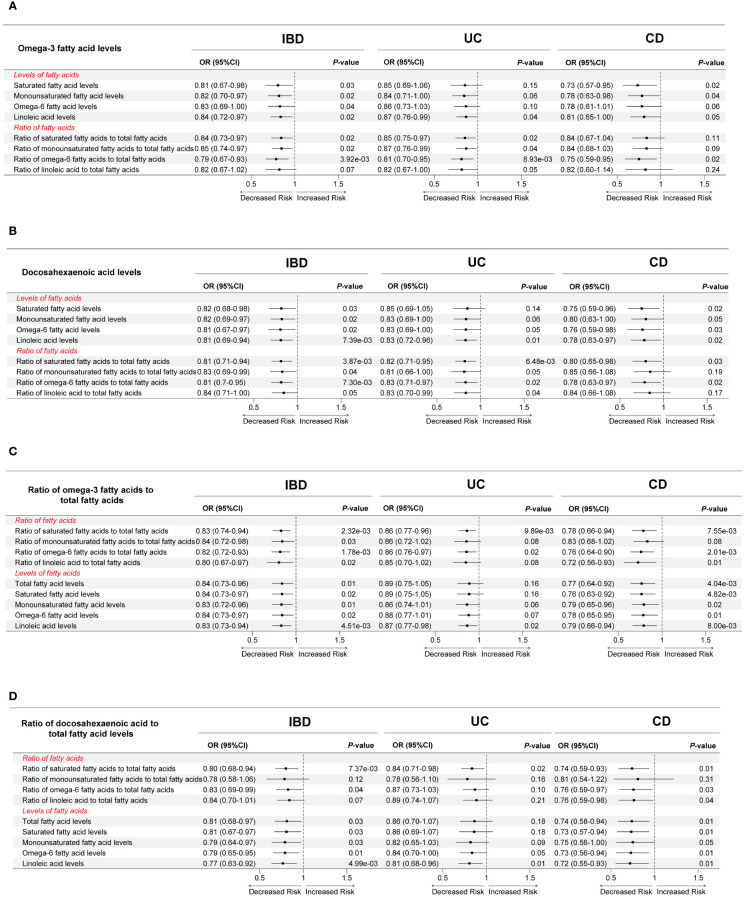
The influence of independently co-existing fatty acid indicators on the causal effect between **(A)** Omega-3 fatty acid levels, **(B)** Docosahexaenoic acid levels, **(C)** Ratio of omega-3 fatty acids to total fatty acids, as well as **(D)** Ratio of docosahexaenoic acid to total fatty acid levels and inflammatory bowel disease and its two subtypes using multivariate Mendelian randomization. OR, odds ratio; CI, confidence interval; IBD, inflammatory bowel disease; UC, ulcerative colitis; CD, Crohn’s disease.

To probe the influence of concurrently co-existing FA indicators on MR results, we utilized the MVMR method to further refine our findings (**Supplementary Table S7**). **Figure 4** visualized the effect of potential FA indicators on the causal relationship between Omega-3 FA levels, DHA levels, Ratio of Omega-3 FA to total FA, as well as Ratio of DHA to total FA and IBD, UC, and CD. First, when using Omega-3 FA levels as exposure, Ratio of LA to total FA affected the association of Omega-3 FA levels with IBD, SFA levels, MUFA levels, and Omega-6 levels influenced the correlation of Omega-3 FA levels with UC, and Omega-6 levels, Ratio of SFA to total FA, Ratio of MUFA to total FA, and Ratio of LA to total FA impacted the relevance of Omega-3 FA levels to CD (**Figure 4A**). Second, when considering DHA levels as exposure, no independent FA indicators affected the association of DHA levels with IBD, whereas SFA levels as well as MUFA levels influenced the correlation of DHA levels with UC, meanwhile, both Ratio of MUFA to total FA and Ratio of LA to total FA impacted the relationship of DHA levels with CD (**Figure 4B**). Third, when Ratio of Omega-3 FA to total FA was used as exposure, the association of Ratio of Omega-3 FA to total FA with IBD was not affected by any of the independent FA indicators, whereas Ratio of MUFA to total FA, Ratio of LA to total FA, total FA levels, SFA levels, MUFA levels, and Omega-6 FA levels influenced the correlation of Ratio of Omega-3 FA to total FA with UC, and Ratio of MUFA to total FA impacted the relationship of Ratio of Omega-3 FA to total FA with CD (**Figure 4C**). Finally, when employing Ratio of DHA to total FA as exposure, Ratio of MUFA to total FA as well as Ratio of LA to total FA levels affected the association of Ratio of DHA to total FA with IBD, whereas Ratio of MUFA to total FA, Ratio of Omega-6 FA to total FA, Ratio of LA to total FA, total FA levels, SFA levels, and MUFA levels influenced the correlation of Ratio of DHA to total FA with UC, and Ratio of MUFA to total FA impacted the relationship of Ratio of DHA to total FA with CD (**Figure 4D**).

**Supplementary Figure S2** depicted the effect of different FA indicators on the association between the two MUFA indicators and CD. First, the MVMR results hinted that the causal relationship between Ratio of MUFA to total FA and CD might be influenced by both Ratio of PUFA to total FA, Ratio of Omega-3 FA to total FA, Ratio of DHA to total FA, Ratio of Omega-6 FA to Omega-3 FA, Omega-3 FA levels, and DHA levels. Additionally, there were no independent FA indicators that would affect the causal effect between Ratio of PUFA to MUFA and CD.

### 3.4 Influence of Genetic Susceptibility to IBD on FA

Given that reverse causation may compromise the accuracy of the results, we further explored the causal associations of genetic susceptibility to IBD, UC, and CD with different FA indicators (**Supplementary Table S8**). We discovered that genetically predicted IBD was associated with increased Ratio of SFA to total FA (IVW: OR = 0.99, *P* = 0.011). Furthermore, there was no causal relationship between genetically predicted IBD, UC, and CD and the remaining FA indicators.

## 4 Discussion

In this study, we used bidirectional two-sample MR analysis to comprehensively investigate the potential causal effects between 17 FA indicators (including different FA levels and ratios) and IBD (including UC and CD). The results of the research indicated that Omega-3 FA levels, Ratio of Omega-3 FA to total FA, DHA levels, and Ratio of DHA to total FA were able to reduce the risk of developing IBD, UC, and CD. Meanwhile, multiple additional MR methods, validation of testing cohort, and merging of meta-analysis ensured the reliability and consistency of these results. Furthermore, the risk effects of Omega-3 FA and DHA for IBD were barely moderated by independent co-existing FA indicators, whereas their causal effects for UC and CD were primarily influenced by SFA and MUFA, respectively. Finally, the conclusions regarding the causal effects between Ratio of MUFA to total FA or Ratio of PUFA to MUFA and CD were not robust. There was no reverse causality for any of the above findings.

The western diet featured a high fat intake (33). There was proof linking a higher fat diet to the etiology of IBD (33). Excessive fat diets might change the gut microbiota’s composition and compromise the integrity of the gut barrier, which can lead to inflammation in the intestines and throughout the body, according to numerous studies (11). Analogous to carbohydrates and amino acids, patients with IBD have been reported to have aberrant fatty acid patterns and metabolism in their blood and intestinal mucosa (34). Moreover, dietary supplementation with specific FA could alleviate disrupted FA profiles and intestinal inflammation (35). Currently, many observational studies have inquired about the relationship between FA and IBD. SFA primarily occurred in products embodying animal fats, such as butter, meat, full cream milk, and other dairy foods. Despite the fact that small case-control studies have shed light on the connection between SFA and IBD risk, prospective cohorts have yet to ascertain the statistical association between them, indicating that the relationship was complicated (11). Moreover, the Mediterranean diet comprising high levels of MUFA, especially oleic acid, has been epidemiologically acknowledged for its salutary role in cardiovascular health and metabolic syndrome (36). The impact of MUFA on IBD remains unresolved as well. Several contradictory statements have been reported regarding the therapeutic potential of MUFA in IBD. A largescale prospective cohort illustrated that dietary oleic acid intake was negatively linked to the development of UC (37). In contrast, other research has shown that high intake of MUFA increased the risk of UC and CD (38). These negative or conflicting findings might stem from the limitations of observational studies, which have created a situation where the causal relationship between FA and IBD continues to be uncertain. Therefore, our MR analysis was able to bridge the gap of previous studies and offer some practical insights into the use of dietary FA to go for prevention and treatment in IBD patients.

Prior studies have demonstrated that Omega-3 FA, represented by DHA, were closely related to the prevention and treatment of IBD. DHA was directly or transcellularly processed to derive hydroxyl-containing Omega-3 FA metabolite (39), yielding potent anti-inflammatory and immunomodulatory properties, which might enhance intestinal mucosal recovery and relieve active symptoms for IBD (40). Such intake of DHA-rich Omega-3 FA was accomplished by consuming oily fish on a regular basis or supplementing with olive oil and fish oil. One study showed that patients with higher dietary DHA intake had a 77% lower risk of UC (41). Moreover, a health study cohort showed that long-term high intake of Omega-3 FA was associated with a reduced risk of UC (12). Regarding CD, a study that enrolled children newly diagnosed with CD found that fish consumption in the year prior to diagnosis was associated with a lower risk of developing the disease (42). Furthermore, a cohort study published in 2014 reported a negatively correlated biological gradient between increased dietary DHA intake and the occurrence of CD (43). Nonetheless, there were some ambivalent results. For instance, a Japanese case-control study demonstrated that excessive ingestion of Omega-3 FA was positively correlated with the risk of CD (44). With the exception of cohort studies, most previous researches have been undertaken in a small number of patients. In the intervention studies, other potential biases that may be raised were consumption patterns, type of food, or type of formula employed. All of these factors seriously confused the results and prohibited reliable comparisons. Consequently, our study proposed a plausible way to explore causal effects, namely MR analysis. The results revealed substantial benefits of Omega-3 FA as well as DHA in lowering the risk of IBD, UC, and CD. Such findings might have critical clinical implications. Hence, healthcare professionals could consider advising people at high risk of developing IBD, especially those with genetic susceptibility, to augment their intake of foods or supplements rich in Omega-3 FA as well as DHA. Nevertheless, it was necessary to note that the results of this study were rooted in genetic analysis, and further studies were required to affirm the clinical utility of these recommendations.

FA regulated the intestinal inflammatory response by affecting the immune system in a variety of ways, such as production of anti-inflammatory and pro-inflammatory mediators, alteration of intracellular lipids, and activation of nuclear receptors (45). Omega-3 FA were tightly linked to inflammation suppression. First, the immunomodulatory properties of Omega-3 FA were connected to the generation of biologically active fat derivatives. Omega -3 FA downregulated the production of pro-inflammatory molecules such as leukotrienes, prostaglandins, and lectins, thereby governing the inflammatory response (46). Second, Omega-3 FA and DHA inhibited COX-2 and ERK phosphorylation as well as IL-17 secretion by Th17 cells, resulting in diminished STAT-3 phosphorylation (47). STAT-3 dephosphorylation attenuated intestinal inflammation. Furthermore, other FA exerted vital functions in the pro-inflammatory procedures of the organism. Traditionally, Omega-6 FA, represented by LA, has been viewed as pro-inflammatory. LA were the primary PUFA in the diet that could be converted to AA, the precursor of inflammatory mediators such as prostaglandins and leukotrienes. Additionally, SFA interplayed with TLR by adding COX-2 expression and ERK phosphorylation (48). They also mobilized other pro-inflammatory pathways of the NLRP3 inflammasome related to cytokine production, such as IL-1β and IL-18 (49). Thus, this might partly explain why the presence of SFA had some influence on the protective effects of Omega-3 FA and DHA on UC.

There have been some previous MR studies to explore the causal association between FA and IBD, including the studies by Jia et al. (50), Astore et al. (51), and He et al. (52). However, our study has its unique strengths and differs from other MR investigations. First, our study incorporated the latest summary data from GWAS on dietary lipids. Our FA data were derived from studies in 2022, whereas other MR studies had utilized data from the United Kingdom Biobank in 2018 or earlier studies in 2011. Second, our research included a comprehensive range of FA types, encompassing total FA levels, SFA levels, MUFA levels, PUFA levels, Omega-3 FA levels, Omega-6 FA levels, DHA levels, and LA levels. In contrast, studies by Astore et al. (51) and Jia et al. (50) only included a more limited scope of FA types. Third, besides FA types, we also comprised ratios between different FAs in our analysis, which helped mitigate interference between different FAs, reducing confounding and bias, thereby enhancing the reliability and accuracy of our study conclusions. Other MR studies did not cover such exposure phenotypes for different FA ratios. Fourth, our analytical methods were more comprehensive and diverse, employing multiple sensitivity analysis techniques such as MR-PRESSO, MR-RAPS, testing cohort, and meta-analysis, ensuring the credibility of our study findings. Additionally, we used MVMR to explore potential interactions or effects between different FAs, further bolstering the reliability of our conclusions. We also conducted FDR correction for multiple testing and performed reverse MR analysis, which we believed other studies did not implement. Other MR studies typically used single and traditional methods, which might not provide as strong evidence and convincing power as our study. Finally, our study specifically emphasized that Omega-3 FA, represented by DHA, could reduce the risk of IBD and its subtypes. In contrast, conclusions from other studies might vary. The research by Jia et al. (50) suggested the causal relationship between elevated EPA levels and reduced risk of IBD and CD but with a weaker impact on UC. Moreover, Astore et al. (51) identified protective effects of Omega-3 FA against IBD without further subtype analysis for CD and UC. He et al. (52) proposed the causal link between Omega-3 FA and UC but not CD. Given these limitations in previous research, we argued that our findings better elucidated the causality between FA and IBD and its subtypes.

However, our study had some limitations. First, the dependence on GWAS data included only individuals of European origin, which precluded the possibility of generalizing our findings to other ethnic populations, thus restricting the cultural diversity dimension of the research. Second, we observed heterogeneity in most of the results. Nonetheless, random effects IVW was still the predominant analysis method, which could effectively keep the combined heterogeneity of the data. At the same time, we have applied a variety of methods to perform iterative validation to ensure the robustness of the conclusions. Third, MR methods were unable to evaluate the nonlinear relationship between exposures and outcomes. Fourth, our research on FA categories was not yet very complete. Other types of FA, such as AA, EPA, or DPA, may warrant further study using more suitable databases.

## 5 Conclusion

This MR study systematically elucidated the causal relationship between 17 FA indicators and IBD and its subtypes (UC and CD). We revealed that Omega-3 FA as well as DHA were effective in reducing the risk of developing IBD, UC and CD. Our findings emphasized the importance of complementing specific FA, particularly in individuals with the genetic susceptibility to IBD. Further studies are needed to corroborate these discoveries and to investigate the potential mechanisms.

## Data availability statement

The original contributions presented in the study are included in the article/**Supplementary Material**. Further inquiries can be directed to the corresponding author/s.

## Ethics statement

Ethical approval was not required for the study involving humans in accordance with the local legislation and institutional requirements. Written informed consent to participate in this study was not required from the participants or the participants' legal guardians/next of kin in accordance with the national legislation and the institutional requirements.

## Author contributions

YZ: Conceptualization, Data curation, Formal analysis, Investigation, Methodology, Visualization, Writing – original draft. ZZ: Conceptualization, Funding acquisition, Project administration, Supervision, Validation, Writing – original draft, Writing – review & editing.
