# Supplementary material for: Unraveling the causal link: fatty acids and inflammatory bowel disease
Source: Front Immunol. 2024 Jul 25;15:1405790. doi: 10.3389/fimmu.2024.1405790 (PMC11306040; doi:10.3389/fimmu.2024.1405790)
Supplement: Supplementary file 1 [file DataSheet_1.docx]

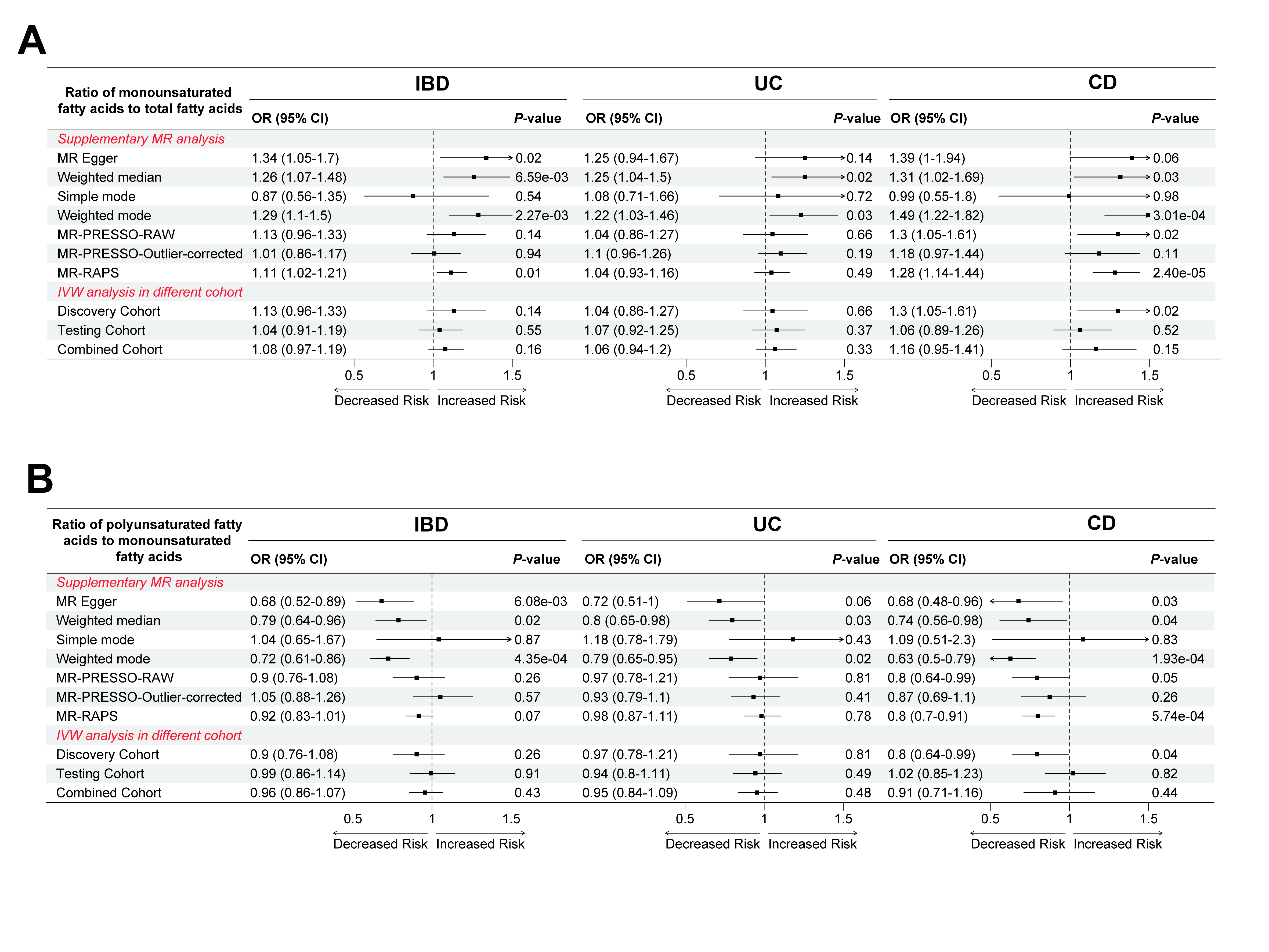


**Figure S1** The causal relationship between (A) Ratio of monounsaturated fatty acids to total fatty acids or (B) Ratio of polyunsaturated fatty acids to monounsaturated fatty acids and inflammatory bowel disease and its subtypes using multiple sensitivity analysis methods. IBD, inflammatory bowel disease; UC, ulcerative colitis; CD, Crohn's disease; OR, odd ratio; CI, confidence interval; MR, Mendelian randomization; PRESSO, Pleiotropy Residual Sum and Outlier; RAPS, robust adjusted profile score.


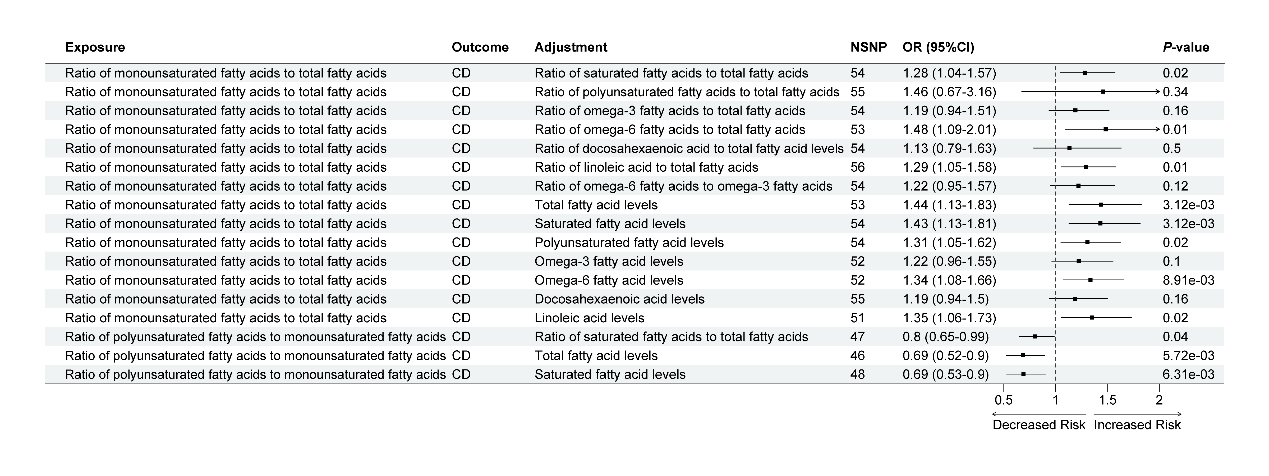


**Figure S2** Multivariate Mendelian adjustment analysis of the causal relationship between two monounsaturated fatty acid indicators and Crohn's disease. CD, Crohn's disease; OR, odd ratio; CI, confidence interval; NSNP, number of single nucleotide polymorphism.
